# Effects of Cerebellar Transcranial Direct Current Stimulation in Bilingual Logopenic Primary Progressive Aphasia

**DOI:** 10.3233/ADR-240034

**Published:** 2024-09-18

**Authors:** Silke Coemans, Vânia De Aguiar, Philippe Paquier, Kyrana Tsapkini, Sebastiaan Engelborghs, Esli Struys, Stefanie Keulen

**Affiliations:** a Brussels Centre for Language Studies (BCLS), Vrije Universiteit Brussels (VUB), Brussels, Belgium; b Groningen Center for Language and Cognition (CLCG), University of Groningen, Groningen, The Netherlands; c Department of Radiation Oncology, University of Groningen, University Medical Center Groningen, The Netherlands; d Center for Neurosciences (C4N), Vrije Universiteit Brussels (VUB), Brussels, Belgium; e Department of Neurology, Johns Hopkins School of Medicine, Baltimore, MD, USA; f Department of Cognitive Science, Johns Hopkins University, Baltimore, MD, USA; g Department of Neurology, Universitair Ziekenhuis Brussel (UZ Brussel), Brussels, Belgium; h Department of Biomedical Sciences, Universiteit Antwerpen (UA), Antwerp, Belgium

**Keywords:** Alzheimer’s disease, bilingualism, cerebellum, executive functions, primary progressive aphasia, transcranial direct current stimulation

## Abstract

**Background:**

Primary progressive aphasia (PPA) is a language-based dementia, causing progressive decline of language functions. Transcranial direct current stimulation (tDCS) can augment effects of speech-and language therapy (SLT). However, this has not been investigated in bilingual patients with PPA.

**Objective:**

We evaluated the case of Mr. G., a French (native language, L1)/Dutch (second language, L2)-speaking 59-year-old male, with logopenic PPA, associated with Alzheimer’s disease pathology. We aimed to characterize his patterns of language decline and evaluate the effects of tDCS applied to the right posterolateral cerebellum on his language abilities and executive control circuits.

**Methods:**

In a within-subject controlled design, Mr. G received 9 sessions of sham and anodal tDCS combined with semantic and phonological SLT in L2. Changes were evaluated with an oral naming task in L2, the Boston Naming Task and subtests of the Bilingual Aphasia Test in in L2 and L1, the Stroop Test and Attention Network Test, before and after each phase of stimulation (sham/tDCS) and at 2-month follow-up.

**Results:**

After anodal tDCS, but not after sham, results improved significantly on oral naming in L2, with generalization to untrained tasks and cross-language transfer (CLT) to L1: picture naming in both languages, syntactic comprehension and repetition in L2, and response times in the incongruent condition of the Attention Network Test, indicating increased inhibitory control.

**Conclusions:**

Our preliminary results are the first to indicate that tDCS applied to the cerebellum may be a valuable tool to enhance the effects of SLT in bilingual patients with logopenic PPA.

## INTRODUCTION

### Primary progressive aphasia

The syndrome of primary progressive aphasia (PPA) is a progressive language disorder caused by neurodegenerative brain disease. The patient’s clinical profile is characterized by a severe language deterioration in the initial stage, with the language impairment being the first characteristic exerting negative effects on daily living activities and other cognitive complaints, including memory failure and executive dysfunctions, occurring more prominently only with disease progress. The current diagnostic criteria classify PPA into three main clinical variants. Classification is based on the most frequently occurring speech and language symptoms and patterns of brain atrophy: non-fluent/agrammatic variant PPA (NfvPPA), semantic variant PPA (SvPPA), and logopenic variant PPA (LvPPA), with the latter type being the clinical variant relevant to this case report (see Table 1). Not all patients can be captured by these criteria, unfortunately, and those cases are usually referred to as ‘unclassifiable’ or ‘mixed’ PPA.^1^ PPA can be caused by variable pathologies and is most often associated with one of three classes of pathology: tau-positive or ubiquitin/TDP-43-positive frontotemporal lobar degeneration, or, as in this case report, Alzheimer’s disease (AD).^2^ In some cases, a particular pathology is associated with a specific PPA variant (see Table 1), although frequent exceptions exist, and there is no one-to-one relationship between pathology and PPA variant.^3^

**Table 1:** Classification of primary progressive aphasia variants

	NfvPPA	LvPPA	SvPPA
Clinical features	•Effortful, halting speech (e.g., apraxia of speech)	•Impaired single-word retrieval in spontaneous speech and naming	•Impaired confrontation naming
	•Agrammatism in language production	•Impaired repetition of sentences and phrases	•Impaired single-word comprehension
	Additionally, at least two of the following features must be present:	Additionally, at least three of the following features must be present:	Additionally, at least three of the following features must be present:
	•Impaired comprehension of syntactically complex sentences	•Phonologic errors in spontaneous speech and naming	•Impaired object knowledge, particularly for low-frequency items
	•Spared single-word comprehension	•Spared single-word comprehension and object knowledge	•Surface dyslexia or dysgraphia
	•Spared object knowledge	•Spared motor speech	•Spared repetition
		•Absence of outspoken agrammatism	•Spared speech production
Neuroimaging*	•Atrophy is most prominent in the left posterior fronto-insular region	•Atrophy is most prominent in the left posterior perisylvian or parietal region	•Atrophy is most prominent in the anterior temporal lobe
Most commonly associated pathology	•FTLD-tau (52%)^152^	•AD (50–60%)^152^	•FTLD-TDP (69–83%)^2^

PPA, primary progressive aphasia; LvPPA, logopenic/phonological variant PPA; SvPPA, semantic variant PPA; NfvPPA, non-fluent/agrammatic variant PPA; AD, Alzheimer’s disease; FTLD-TDP-43, frontotemporal lobar dementia with ubiquitin and transactive response DNA binding protein kDa (TDP-43) pathology; FTLD-tau, frontotemporal lobar dementia with tau-positive pathology; ^*^Disease epicenters. Pathology progresses and atrophy may become more widespread, including white matter and functional connectivity alterations.^153–160^

### Bilingual primary progressive aphasia

As our society of globalization becomes more and more bilingual, the number of people with PPA who are bilingual by inference also increases. Compared to the more extensively investigated monolingual aphasia, bilingual aphasia comes with some peculiarities that need to be considered. Bilingualism is mediated by structural and functional plastic changes in the brain, leading to neural differences between bi-and monolinguals: the mother tongue (L1) and second language (L2) share neural substrates to some extent (e.g., Mechelli et al.^4^ Ressel et al.^5^), but the use of L2 engages regions outside of the ‘traditional’ left hemisphere language areas (e.g., Wartenburger et al.^6^ Pliatsikas et al.^7^). These regions are associated with controlling the use of the target language in a given conversational context, without interference of the non-target language (bilingual language control).^8–10^ This mechanism of bilingual language control is associated with the use of a complex set of mechanisms for inhibitory control.^11^ Several factors, distinct but interacting, are thought to influence the different cortical representations of language in bilinguals, including the age of acquisition, L2 proficiency and L1/L2 exposure.^12,13^

Research on bilingual PPA is scarce and is predominantly focused on patterns of language decline. Most published articles are case studies, which often report earlier or larger decline in L2,^14–23^ although some also report parallel decline.^24–26^ In a retrospective group study, deficits in the LvPPA group appeared largely parallel when comparing L1 and L2 along the language domains.^27^ The largest study to date on bilingual PPA investigated 16 SvPPA patients, who had substantial worse impairment in L2 compared to L1,^28^ contrasting the parallel decline more often found in the other variants in previous studies.^27^

Knowledge on which language may be better preserved or more affected in bilinguals with PPA is pivotal for tailoring interventions effectively. However, there is a notable gap in research when it comes to treatment approaches for bilingual aphasia, even more so in the context of PPA. Questions arise regarding whether treatment should target one language specifically or both, and whether cross-language transfer (CLT) of treatment effects might occur. Currently, formal assessment measures and clear guidelines for evaluating bilingual PPA are lacking, and speech-and language therapy (SLT) intervention in bilingual PPA largely unexplored.^29^ So far, only two treatment studies are published on bilingual PPA. In a case-study, a Norwegian (L1)-English (L2) woman with LvPPA received phonological and orthographic naming treatment in L2, over a one-year time period, with approximately three sessions per week.^30^ Forty out of the 60 trained stimulus words were Norwegian-English cognates. Orthographic treatment led to greater L2 written naming accuracy and CLT to her L1. The authors proposed that the high proportion of cognates in the study facilitated performance in the untrained written naming condition. The phonological treatment resulted in better L2 oral naming results, but without CLT. Further, in a case series of 10 bilingual speakers with anomia, participants received lexical retrieval treatment, with one language treated per phase, each phase consisting of nine sessions over five weeks. Individually tailored word sets had to be named via guided retrieval of semantic, phonological and orthographic information (lexical retrieval cascade). Equal numbers of cognate and noncognate words were included to evaluate their potential to facilitate CLT. Participants showed a significant effect of treatment in each of the treated languages. There were instances of CLT: mostly for cognates, but also significantly for noncognates. Languages spoken by the patients varied in combinations, but the number of participants was too low to hypothesize on influences of structural similarities across the languages.^31^

Given the scarcity of research in PPA, we briefly elaborate on studies on bilingual post-stroke aphasia to inform our understanding. Importantly, the recent meta-analysis by Goral et al. (2023) provided evidence that treatment in either language can lead to recovery, often with the greatest effects observed in the treated language (as in^32,33^). Furthermore, significant cross-language generalization effects were identified, indicating that language therapy in one language could benefit untrained languages, suggesting a potential benefit of treatment for bilingual individuals with aphasia.^34^ While there is no clear consensus on the variables contributing to CLT, factors such as: pre-and post-morbid language proficiency, structural similarities between languages, word type (cognate/noncognate) and cognitive control have been proposed in post-stroke literature.^33,35,36^

Given these findings, this case-study aims to further elucidate whether similar cross-language effects can occur in a patient with bilingual LvPPA and explore the potential role of neurostimulation in facilitating these effects (see below).

Interestingly, executive functions have yet to be explored in patients with bilingual PPA, despite research interest in a potential relationship between executive functions and bilingualism. ‘Executive functions’ are a multidimensional construct of higher-order, general purpose control mechanisms, that control and regulate lower-level cognitive processes and independent, goal-directed behavior.^37^ According to the seminal model of Miyake et al.^38^ there are three established components of executive functions: inhibition of prepotent responses (inhibition), shifting between mental states (shifting), and updating and monitoring of the working memory (updating). Executive functions and language have been suggested to have a reciprocal relationship. For example, in post-stroke aphasia, patients can exhibit impaired performance on tasks related to all three executive functions, correlating with their degree of language deficits.^39,40^ In bilingual speakers, there has been growing interest in whether bilingual individuals use executive function-like processes during bilingual language control, and to which extent this control is exerted by engaging a network of cortical and subcortical brain regions closely related to executive function.^8,41–44^ Deficits of bilingual language control have been linked to pathological switching and mixing of the languages.^45,46^ Further, in post-stroke aphasia, bilingual language control has been proposed as a contributing factor to the patterns of decline and recovery, as well as possibility of CLT in bilingual aphasia.^8,47^ For example, Green^48^ proposed that the inability to produce both languages simultaneously may be due to a deficit in bilingual language control. A lively debate exists on whether the nature of bilingual language control is domain-general or domain-specific, that is, whether it involves general cognitive control (i.e., executive functions), or control specific to linguistic mechanisms.

In post-stroke aphasia, two recent review articles found evidence for involvement of a domain-general cognitive control mechanism as well as involvement of a domain-specific language control mechanism.^43,49^ Exploring executive functions in (bilingual) PPA can add to this ongoing debate and provide nuance to the perspective when considering patterns of interaction between executive functions and language in patients with aphasia. One study so far has studied executive functions in multilingual individuals with SvPPA. Their findings revealed that while the patient experienced a semantic storage deficit, typical in SvPPA, she had more difficulty accessing word meanings in a non-dominant language, indicating an interaction between conceptual degradation and language-specific lexical retrieval processes.^50^ One possible reason why executive functions have been overlooked in this population so far, is the historical assumption that they are not significantly impaired by PPA, coupled with limited investigation in monolingual PPA samples.^2^ Recent meta-analyses however indicate that deficits of executive functions can exist in all clinical variants.^51–53^

### Transcranial direct current stimulation in PPA

There are currently no disease-modifying drugs available to stop the progression of the neuropathologies underlying PPA, and SLT is the main option for symptomatic treatment of language impairments in these patients. Transcranial direct current stimulation (tDCS) is a tool for neuromodulation that has been under investigation to enhance the effects of SLT. tDCS applies a low-intensity current to the scalp, using an anodal and a cathodal electrode, and can be used to increase (excite) or decrease (inhibit) neuronal excitability under the stimulated area.^54^ Often, anodal tDCS stimulation is thought to cause excitation and cathodal tDCS to cause inhibition, although this dual-polarity effect is not always found.^55^ Recent meta-analyses of effects of tDCS combined with SLT in PPA, found significant larger effects for naming therapy (oral and written) combined with anodal tDCS as compared to sham (placebo) stimulation.^56–58^

The most often applied methodology so far has been anodal tDCS over the left inferior frontal gyrus, combined with oral and/or written naming therapy as noted in a recent review by our group.^59^ There is, however, quite a lot of heterogeneity in the settings of different tDCS parameters (e.g., site(s) of stimulation, stimulation frequency, type of combined SLT, etc.), and not all cortical sites of stimulation have benefitted all PPA variants, and not all variants seem to benefit the same from tDCS.^60^ See Table 2 for an overview of the main findings of our systematic review on tDCS in PPA.^59^ This makes clinical translation for tDCS difficult, and the search for optimal stimulation parameters is still ongoing. One question that pertains for instance, is whether tDCS should be applied to areas of atrophy, or to less affected structures. A compelling argument can be made for stimulating less degenerated regions to capitalize on their intact functionality, potentially compensating for deficits in the primary language areas,^59^ such as for instance the cerebellum.

**Table 2:** General findings systematic review (Coemans et al.^59^)

Protocol similarities	Protocol differences
Stimulation duration: 20–30 min	Stimulation session frequency:
	•1–5 sessions/week over 2-3 weeks
Stimulation intensity: 1-2 mA	Type of language therapy:
	•none (*n* = 4)
	•oral naming (*n* = 5)
	•oral and written naming (*n* = 5)
	•word fluency (*n* = 1)
	•word repetition (*n* = 2)
Electrode surface: 5×5 cm or 5×7 cm	Location of stimulation (all in left hemisphere):
	•inferior frontal gyrus (IFG, *n* = 9)
	•inferior parietal lobe (IPL, *n* = 1)
	•dorsolateral prefrontal cortex (DLPFC, *n* = 2)
	•IPL versus IFG (n 1)
	•IPL versus DLPFC (*n* = 1)
	•temporal; anodal versus cathodal (*n* = 1)
	•perisylvian and Broca’s area (*n* = 1)
Patient characteristic similarities	Patient characteristics differences
Mean age: 66 (SD: 6.3) to 68.7 (SD: 7.0) years old	Composition of study population:
	•mixed group (*n* = 10)
	•NfvPPA only (*n* = 5)
	•SvPPA only (*n* = 1),
	•LvPPA only (*n* = 1)
Average disease duration: 4.9 (SD: 0.9) years	Total *n* participants per PPA variant:
	•110 NfvPPA
	•62 LvPPA
	•52 SvPPA
Disease severity: mild to moderate
Summary language outcome measure results
•Despite protocol heterogeneity, 16 out of 17 studies report positive tDCS-related language outcomes.	
•These outcomes are variable regarding size, duration, and generalization of effects.	
•Not all PPA variants benefit from all protocols.	
•Stimulation location and clinical variant are determinants of tDCS effects.	

PPA, primary progressive aphasia; LvPPA, logopenic/phonological variant PPA; SvPPA, semantic variant PPA; NfvPPA, non-fluent/agrammatic variant PPA.

### Cerebellar tDCS in bilingual aphasia

In the field of bilingual PPA, however, research on tDCS remains scarce. Again, insights from post-stroke studies may help shed light on potential stimulation targets. Two publications of neurostimulation are published to date, both being case studies of patients who sustained a left frontal ischemic stroke. The first study applied transcranial magnetic stimulation to stimulate the dorsolateral prefrontal cortex in a patient presenting with pathologic language switching.^61^ Inhibitory stimulation increased language switching, while excitatory stimulation stopped language switching. The second publication is from our research group.^62^ We applied anodal tDCS to the cerebellum in a bilingual patient with severe non-fluent aphasia, finding improvements in oral picture naming in the treated language (L2) after tDCS and sham, with generalization to untrained items and the untreated language (L1) only after real tDCS. Picture naming score improvement was mainly related to a reduction in semantic errors and omissions.

The cerebellum emerges as a novel candidate stimulation location due to its relatively spared nature in PPA.^63,64^ White matter tract-tracing studies have revealed crossed anatomical connections between the lateral cerebellar hemispheres and frontal and parietal association areas in the contralateral cerebral cortex, including regions involved in language processing, executive functions and (bilingual) language control.^65–69^ Through these white matter pathways, tDCS may be able to engage the residual left cortico-subcortical hemispheric network and facilitate information exchange between the cerebellum and cortical regions. Specifically, cerebellar tDCS is believed to influence Purkinje cell polarization, thereby altering activity levels in the deep cerebellar output nuclei.^70^ Consequently, this modulation impacts excitability and plasticity in distant cortical areas,^54,71^ potentially facilitating rehabilitation.^72,73^ Further, fMRI studies show activation of the right posterolateral cerebellum during a plethora of tasks related to language processing,^74–81^ and executive functions.^81–84^ Conversely, lesion studies associate damage to the right cerebellum with deficits language, at times giving rise to cerebellar-induced aphasia (for a review, see De Smet et al.^68^). Further, improved outcome of aphasia rehabilitation has been associated with increased activation in the right cerebellum.^85^ In bilingual individuals with PPA, who must manage two linguistic systems, the cerebellum’s function in coordinating cognitive resources during language processing becomes particularly relevant.^44,86–88^ Additionally, considering the cerebellum’s connections with the basal ganglia, stimulating the cerebellum may indirectly modulate activity in these structures. These effects may contribute to improvements in language-related tasks by enhancing procedural learning and cognitive control mechanisms mediated by the basalganglia.^69,89^

Existing evidence suggests that stimulation over the right posterolateral cerebellum in healthy adults has improved verbal fluency and increased functional connectivity between the cerebellum and motor speech areas.^90^ In post-stroke aphasia, cerebellar tDCS has shown efficacy in enhancing effects of different tasks trained during speech and language therapy.^62,72,91,92^

Given the dearth of research on bilingual PPA, we aspired to evaluate whether previous findings also hold for this population. Further, tDCS applied to a relatively spared distant, but structurally connected node of the language network, might be applicable to different PPA patients with a variety of regions of atrophy. This research may also help provide insights into the role of the cerebellum in language.

**Fig. 1:**
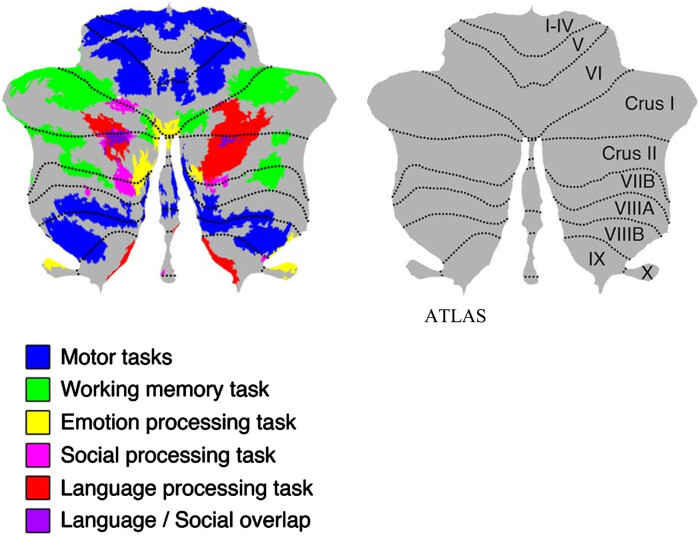
Topographic organization cerebellar lobules. The figure displays cerebellar fMRI task activations to language (Crus I/II, region stimulated in our study), cognitive and motor tasks, on a flatmap of the cerebellum. Adapted from Figure 1: Functional gradients of the cerebellum, by Guell et al., used under CC BY 4.0 / Source: *eLife*.^161^

**Fig. 2:**
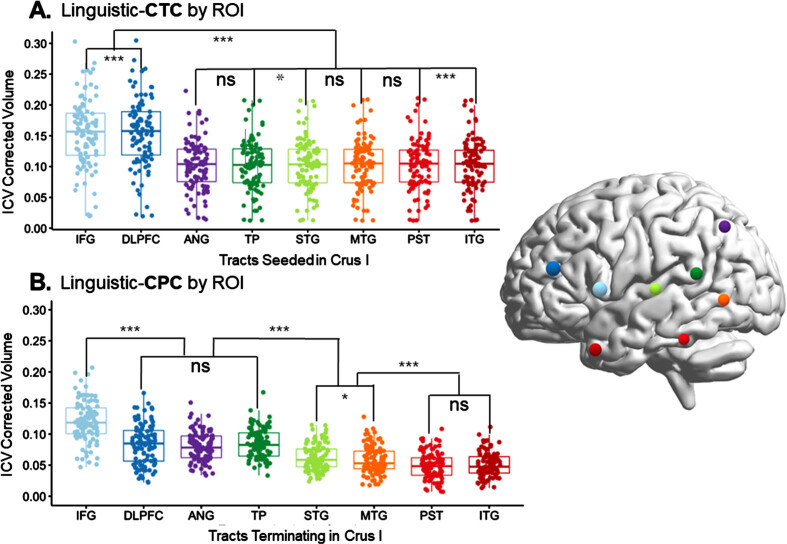
White matter connections right hemisphere cerebellum and left hemisphere cerebral cortex. Reconstruction of white matter pathways connecting regions of interest (ROIs) in the right cerebellum with those in the left cerebral cortex, specifically focusing on the cerebello-thalamo-cortical (CTC) and cortico-ponto-cerebellar (CPC) pathways. The figure presents a comparison of volumes: A. among tracts originating in right Crus I (stimulated in our study) and terminating in various left-lateralized cerebral language targets; and B. among tracts projecting from cerebral language ROIs to Crus I (stimulated in our study). IFG, inferior frontal gyrus; DLPFC, dorsolateral prefrontal cortex; ANG, angular gyrus; PST, posterior superior temporal lobe; STG, superior temporal gyrus; MTG, middle temporal gyrus; ITG, inferior temporal gyrus; TP, temporal pole. Adapted from Figure 3: Language and the cerebellum: structural connectivity to the eloquent brain, by Jobson et al., used under CC BY 4.0 / Source: Neurobiology of Language.^67^

Specifically, our research objectives were as follows:

• Firstly, to provide insights of patterns of bilingual language decline in PPA, to add to the small body of knowledge on this topic.
• Secondly, to assess the impact of tDCS stimulation applied to the right posterolateral cerebellum during SLT on language abilities in a patient with logopenic variant PPA. We hypothesized that cerebellar tDCS could enhance both semantic and phonological processing (both trained during SLT, see below), targeting Crus I/II of the cerebellum, which is connected to all fundamental cortical areas of language processing (see Figs. 1 and 2).^93^
• Thirdly, we aimed to identify the language skills that benefit from cerebellar tDCS and evaluate the generalization of effects to untrained tasks, using subtests of the Bilingual Aphasia Test (BAT).
• Fourthly, to explore whether cerebellar tDCS facilitates CLT in bilingual PPA patients.
• Lastly, to assess whether the executive control circuits benefit from tDCS application and associated language therapy in bilingual PPA, via an attention network test and Stroop test, since crossed cerebro-cerebellar connections (connecting to basal ganglia and (pre-)frontal regions)^67,69^ are neuronally reinforced via stimulation.

## METHODS

### Participant

Mr. G. was a 59-year-old Belgian university professor. The COVID-19 pandemic and its restrictions reduced his social interactions over two years, except for sporadic conversations with family. Upon returning to work, he noticed newfound language difficulties prompting a hospital referral in early 2022. He presented with complaints about what seemed to be hearing problems and difficulty in understanding spoken language. It is difficult to estimate how long the symptoms were present but left unnoticed.

The neuropsychological investigation that led to his diagnosis was conducted one year prior to inclusion in our research. It was concluded that he produced non-fluent and agrammatic speech, with phonological and semantic paraphasias, notable word finding difficulties and problems with naming and articulation. Language comprehension was slightly affected. Further, he showed limitations regarding anterograde memory, more so in episodic than semantic memory, and problems with encoding of visual information. Behaviorally, he exhibited reduced spontaneity and goal orientation, planning difficulties, increased appetite, and attentional challenges with multiple stimuli. However, his visuoconstruction abilities remained intact, with stable mood and sleep patterns, and minimal stressors in his personal and professional life.

MRI showed a slight hippocampal global subcortical atrophy without a clear lobar distribution, while his PET scan showed hypometabolism in the left posterior parietal region. He had a pathological cerebrospinal fluid biomarker profile that was neurochemically characteristic for AD (see Table 3), leading to a diagnosis of AD with logopenic PPA, at the age of 58.

**Table 3:** Neurogenic markers

Marker	Value	Unit	Reference value
Protein Tau concentration	568	+	pg/mL	<403
Protein beta-Amyloid 1–42 concentration	614	–	pg/mL	>722
Protein beta-Amyloid 1–42/1–40	0.062	–	Ratio	>0.07
Phospho-tau concentration	102	+	pg/mL	<50
Protein 14-3-3	Negative

### Language background

Self-reported L1 and L2 proficiency and exposure data was collected via the LEAP-Q questionnaire.^94^ French is Mr. G.’s first language (L1), and was the only language spoken in his child home environment. He acquired Flemish (L2) from the age of twelve, attending a Flemish-speaking secondary school. Education in a new language was difficult at first, and he had to redo his first year. However, he grew to become a very good student, and after secondary school, he continued his education at a Flemish university. Mr. G. reports that during his adult life, he feels like his Flemish was his dominant language. As a researcher and professor, he used English to publish and present his work and to connect with international peers, while he gave his classes in Flemish, and his direct colleagues were mainly Flemish speaking. We can conclude that Mr. G. was a successive, highly proficient speaker of French and Flemish all-around, and English in his professional life. He used French mainly with his family, and Flemish in all communication contexts (on a daily basis), although many of his frequent conversation partners were bilinguals themselves, so he often used multiple languages with the same co-interlocutor within the same conversation. At time of enrollment, his usage had changed: his main person of contact was a family member, with whom he mainly spoke French (L1). He had been receiving speech and language therapy in Flemish (L2).

### Intervention

**Fig. 3:**
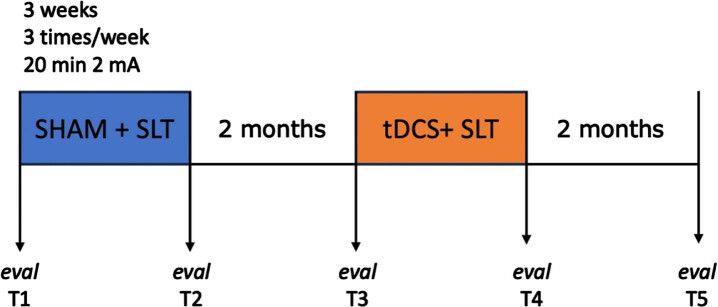
Study design. A within-subject controlled design with pre-and posttreatment evaluations after sham and tDCS was performed. Stimulation was given three times per week, for 3 weeks, during speech and language therapy.

See Fig. 3 for a flowchart of the study design. Mr. G. was treated across two treatment phases, the first being the sham phase, and the second being the tDCS phase. Each phase was preceded by a baseline assessment (T1, sham and T3, tDCS), and followed by a post-treatment assessment (T2 and T4), and a 2 month-follow-up assessment (T3 and T5). Baseline testing in phase 2 is also the follow-up session for phase 1 (T3).

The language therapy was provided in L2, upon the participant’s request, and focused mainly on word retrieval, including an oral picture naming intervention. We assembled a list of 80 therapy picture items (trained items), of which words were matched to items of the untrained Boston Naming Test (BNT) with regard to number of syllables, word frequency, and imageability. The baseline assessment was repeated three times for the naming test, and provided a multiple baseline which was distributed over four days, wherein performance was relatively stable. Pictures that were not named correctly during at least one of three baseline picture naming assessments, were included for therapy, leading to 36 items included for each stimulation phase. These 36 items were different for both stimulation phases. Each item was scored on a four-point scale, as in the Dutch BNT, leading to a maximum possible score of 108 (36 items times three). During naming therapy, Mr. G. was asked to name a picture, and if he was not able to do so within 10 s, the therapist asked him to described features of it, as in semantic feature analysis (e.g., What does it do? What do you use it for? Where can you find it? What does it look like?).^95^ If still not able to name the item, the therapist provided semantic, and additionally phonological, cues to facilitate naming. Lastly, if he could still not name the item, the therapist said the word out loud, and Mr. G. was asked to repeat it. Further, SLT included word-finding tasks, where Mr. G. was for instance prompted to provide a word matching a given definition, or vice versa, thereby targeting lexical retrieval and semantic processing. Further, semantic categorization tasks were employed to enhance his ability to classify words based on their meanings, promoting semantic fluency and organization. Additionally, phonological training exercises, such as syllable reinforcement tasks, were incorporated to improve phonemic awareness and articulatory precision.

Twenty min of tDCS of 2 mA (phase 2) or sham (phase 1) was applied during language therapy, using a direct current stimulation device (Oasis Pro, Mind Alive Inc., Canada), with a 3×3 cm saline-soaked sponge anode placed over the right posterolateral cerebellum (4 cm lateral to the inion and 1 cm down, over right lobule VI), and the cathode placed on the right deltoid muscle.^91^ The patient received sham (no stimulation) and anodal tDCS, in a randomized within-subject controlled design. During the sham tDCS phase, setup was identical to the active tDCS condition, including electrode placement and duration of stimulation. However, unlike active tDCS, the current intensity was ramped up and then immediately down at the beginning and ending of stimulation, to create the sensation of receiving stimulation, without inducing any neuromodulatory effects, thereby serving as control condition. tDCS sessions took place three times a week, for three weeks, for a total of nine sessions per stimulus phase (anodal/sham tDCS). Language therapy lasted for another 10 min after the end of stimulation. A two-month break was introduced in between the two stimulus conditions, to avoid possible interference effects. During this break, speech and language therapy continued as before enrolment in our study: Mr. G. received bi-weekly sessions of 30 min, as per his doctor’s prescription. The patient, therapist and researcher were blinded to the stimulusconditions.

### Behavioral and neurolinguistic assessment

Mr. G. performed assessments of language and other cognitive functions before both tDCS stimulation phases [T1 and T3] for baseline scores, and immediately after three weeks of stimulation [T2 and T4] to evaluate changes in scores after therapy, and at eight weeks follow-up evaluation [T3 (baseline measurement of the second phase is the follow-up measurement of the first phase) and T5]. All baseline languages scores are presented in Table 4.

**Table 4:** Patient characteristics and baseline performances in French (L1) and Dutch (L2)

Age at diagnosis	58 years, 3 months
Age at enrollment	59 years, 5 months
Time since diagnosis	1 year, 2 months
Years of education	20
Mini-Mental State Examination (30)	19
Language background	French (L1)	Dutch (L2)
Age of acquisition	Birth	12
Premorbid proficiency (all modalities, 10)	10	10
Premorbid exposure (100%)	25	50
Postmorbid exposure (100%)	45	40
Bilingual Aphasia Test (232)	172	185
Part B
Verbal auditory discrimination (18)	12	15
Syntactic comprehension (42)	28	36
Repetition of words and nonsense words (30)	20	16
Repetition of sentences (7)	4	3
Verbal fluency - semantic	3	5
Verbal fluency - phonetic	2	2
Semantic Opposites (10)	10	10
Synonyms (5)	3	4
Antonyms (5)	3	4
Reading out loud – words (10)	10	10
Reading out loud – sentences (10)	10	10
Copying (5)	5	5
Dictation /5)	4	4
Reading comprehension – words (10)	7	10
Reading comprehension – sentences (10)	10	10
Sentence construction (32)	28	23
Part C
Grammaticality Judgements	10	9
	L1 ->L2	L2 ->L1
Word Recognition (5)	5	5
Word Translation (10)	5	4
Sentence Translation (18)	8	7
Boston Naming Test (177)	65	97
Personalized Naming Test (108)		42
Attention Network Test
RT congruent trials (ms)	583.95
RT incongruent trials (ms)	660.02
RT incongruent - congruent (ms)	76.07
Stroop task
Interference score (s)	77
*T*-score	40
Percentile	16th

Maximum test scores are listed within brackets. Personalized picture naming consisted of 36 picture items, the Boston Naming Test (BNT) consists of 59 picture items, and on both tests, 3 points per item can be scored.

#### Language measures

To assess his language capabilities in L1 and L2, we performed subtests of the Bilingual Aphasia Test (BAT^96^, L1 and L2), the BNT^97^ (L1 and L2), Picture Description tasks in L1 and L2 from the Comprehensive Aphasia Test-NL^99^ and Boston Diagnostic Aphasia Examination, Cookie Theft Picture,^99^ and an oral object naming assessment (see previous paragraph further explanation), of which the 36 items were scored in the same manner as items of the BNT, where each item is assessed on a four-point scale (0, 1, 2, and 3). Thus, the maximum score that can be obtained on the personalized naming assessment is 108, and on the 59-item BNT, the maximum score is 177. The BNT in Dutch and French contains Dutch-French cognates: 23 out of 59 items (26%). Our study was not designed to evaluate cognate status specifically, but for completeness we add that our personalized naming assessment, had 7/36 (19%) Dutch-French cognate items in the sham phase, and 9/36 (25%) in the tDCS phase. Please note that we attempted to assess the BNT in English as well, but Mr. G. exhibited difficulty in naming items or producing language in that particular language. Consequently, we discontinued the evaluation of English for this individual.

#### Non-linguistic cognitive functioning

To screen for global cognitive functioning, we conducted the Mini-Mental State Examination.^100^ We assessed executive functions, with two tests for inhibition: the non-verbal Attention Network Test (ANT), and a test requiring verbal responses: the Stroop task.^101,102^ A shortened version of the ANT with a total of 144 trials was used to assess the executive network (inhibitory control).^101^ The ANT is a computerized task that combines Eriksen’s Flanker task with Posner’s cuing task, to test attentional networks (the alerting, orienting, and executive network), of which we looked into the executive network.^103,104^ Five black arrows are presented in the middle of the computer screen, and the participant is asked to give a directional response to the middle target arrow. The arrows flanking the target arrow can be congruent (where all arrows point in the same direction), or incongruent (where flanking arrows point in a different direction than the target arrow). The measure of executive function efficiency is then the difference in response times between the incongruent and congruent conditions: the “Flanker effect”. As the incongruent condition presents conflicting information, inhibition is required to elicit a correct response, leading to longer response times than in the congruent condition. A lower Flanker effect indicates greater executive network efficiency. The Dutch adapted version of the original Stroop test consists of three cards (I, II, III), each containing 100 stimuli that have to be read aloud or named as fast as possible.^105^ Card I depicts the names of the colors red, green, yellow, and blue. Card II depicts rectangles in these colors. Card III contains the names of these colors, printed in a non-matching color of ink. Interference occurs when card III requires the participant to name the color instead of reading the word. The raw score is calculated using the formula: time in seconds needed for card III minus time in seconds needed for card II, and based on the guidelines for norming, an interference score and T-score is calculated, corrected for age, education and score card II. ^106^

### Statistical analysis

In both stimulation phases, we compared baseline performance with post-treatment (immediately after three weeks of tDCS) and follow-up (2 months after three weeks of tDCS) The baseline pre-treatment measurement time point of the second phase, was considered to be the follow-up measure time point of the first phase. We used McNemars’ test for correlated responses to assess significance of changes of naming scores and of the subtests of the BAT. We analyzed the Flanker effect (difference in response times of the incongruent and congruent condition in the Attention Network Test) with a repeated measures ANOVA, with “congruency type” (congruent and incongruent condition), “stimulation type” (sham and anodal tDCS) and “timing” (pre- treatment, post-treatment and follow-up) as the within-subject factors. When appropriate, a *post-hoc* correction according to Bonferroni was performed.

## RESULTS

### Baseline performance

See Table 4 for baseline scores. Mr. G. had an MMSE score of 19/30 upon enrollment, with low scores on subtests for memory, attention and language. On the BAT, he had a total score of 172/232 in L1-French and 185/232 in L2-Dutch, with a slightly higher score in L2 on most subtests: verbal auditory discrimination (12/18, L1 versus 15/18, L2), syntactic comprehension (28/42, L1 versus 36/42, L2), semantic fluency (3, L1 versus 5, L2), synonyms and antonyms (both 3/5, L1 versus 4/5, L2), reading comprehension of words (7/10, L1 versus 10/10, L2). He scored better in L1 than L2 on the subtests of repetition of words and nonsense words (20/30, L1 versus 16/30, L2), repetition of sentences (4/7, L1 versus 3/7, L2), and sentence construction (28/32, L1 versus 23/32, L2). For the other subtests, he obtained the same scores for both languages, which were usually maximum scores: semantic opposites (10/10), reading out loud words (10/10) and sentences (10/10), copying (5/5), dictation (4/5), and reading comprehension of sentences (10/10). On BAT Part C, he scored better in L1 than L2 on the grammaticality judgements task (10, L1 versus 9, L2), the word translation task (5/5, L1 versus 4/5, L2), and the sentence translation task (8/18, L1 versus 7/18, L2). On the word recognition tasks, he scored 5/5 in L1 and L2. On the Boston Naming Test, he scored significantly better in L2 (97/177) than in L1 (65/177), *χ*^2^(1, N = 59) = 33.20, *p* < 0.001. On the personalized naming test (L2), he scored 42/108. On the ANT, in the congruent condition, his mean response time was 583.95 ms, and in the incongruent condition 660.02 ms. His raw Stroop interference score was 77 s, which pertains to a T-score of 40, and a performance in the 16thpercentile.^107^

### Effects of cerebellar tDCS on task performance

See Table 5 for results. On the accuracy scores in the personalized naming task in L2, Mr. G. improved significantly after tDCS *χ*^2^(1, N = 36) = 26.70, *p* < 0.001, but not after sham, *χ*^2^(1, N = 36) = 14.73, *p* = 0.59, and tDCS effects lasted until follow-up, *χ*^2^(1, N = 36) = 15.24, *p* < 0.001. Untrained BNT scores improved significantly after tDCS but not after sham, in both the trained (L2, tDCS, *χ*^2^(1, N = 59) = 19.70, *p* < 0.001, sham, *χ*^2^(1, N = 59) = 5.57, *p* = 0.18), and untrained language (L1, tDCS, *χ*^2^(1, N = 59) = 4.33, *p* = 0.04, sham, *χ*^2^(1, N = 59) = 0.18, *p* = 0.67). tDCS results lasted until follow-up in L1, *χ*^2^(1, N = 59) = 8.40, *p* = 0.001.

**Table 5:** Scores language outcomes and executive functions at all assessment time points

Treatment phase	Sham				
	tDCS				
	Pre-treatment	Post-treatment	Two months post-treatment sham / Pre-treatment tDCS	Post-treatment	Two months post-treatment tDCS
T1	T2	T3	T4	T5
Picture naming^1^
Trained items – L2 (/108)	42	59	49	**85**	**69**
Untrained (BNT) - L2 (177)	97	81	77	**104**	97
Untrained (BNT) – L1 (177)	65	65	52	**82**	**69**
BAT^2^
Syntactic comprehension – L2 (51)	36	33	30	**35**	32
Repetition of words and nonsense words – L2 (30)	16	17	12	**20**	18
Attention Network Test^3^
RT congruent trials (ms)	583.95	597.96	605.59	571.17	579.77
RT incongruent trials (ms)	660.02	672.36	697.46	**649.22**	**652.33**
RT incongruent – congruent (ms)	76.07	74.4	91.87	78.05	72.56
Stroop task
Interference score (s)	70	41	47	49	51
*T-*score	40	47	46	49	51
Percentile	16	38	34	31	31

T2 and T3 are compared to T1 (baseline sham phase), with T2 immediately post-treatment and T3 two months post-treatment. T4 and T5 are compared to T3 (baseline tDCS phase), with T4 immediately post-treatment and T5 two months post-treatment. ^1^Maximum test scores (not total number of picture naming items) are listed within brackets. Trained picture naming consisted of 36 picture items per phase (sham/anodal ctDCS), with different items for each phase, and each item scored on a four-point scale (0, 1, 2, or 3). The untrained Boston Naming Test (BNT) consists of 59 picture items. Picture naming items differed in each phase, so McNemar’s test for paired items was not possible to compare T3 with T1 results. ^2^For the Bilingual Aphasia Test (BAT) we only present results with significant change (see Supplementary Table 1 for all scores). ^3^Repeated measures ANOVA comparing response times between pre-treatment and post-treatment. Bold indicates significant change.

BAT syntactic comprehension scores improved significantly after tDCS, *χ*^2^(1, N = 51) = 4.77, *p* = 0.03, but not after sham *χ*^2^(1, N = 51) = 0.47, *p* = 0.32, in the treated (L2), but not in the untreated language (L1). Significance did not last until follow-up. The same is true for the subtest of repetition of words and nonsense words, with after tDCS, *χ*^2^(1, N = 30) = 3.32, *p* = 0.03, and after sham, *χ*^2^(1, N = 30) = 0.11, *p* = 0.37. Note: for the BAT we only present scores with significant changes. See Supplementary Table 1 for all test scores.

**Fig. 4:**
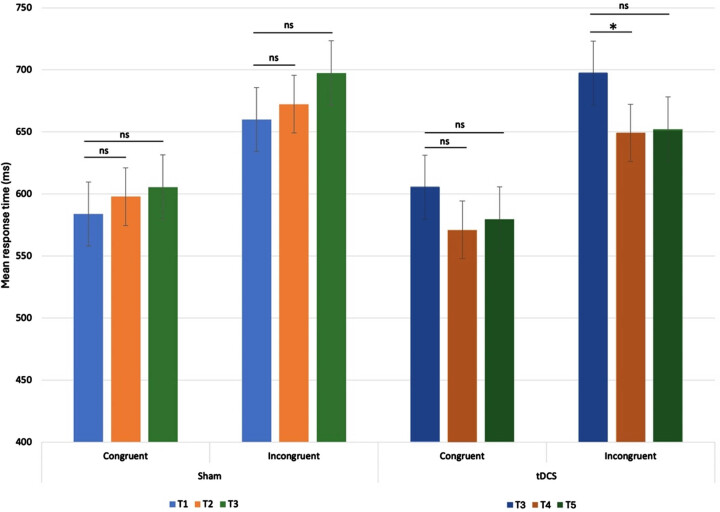
Mean response times Attention Network Test. Mean response times and standard errors per stimulation phase (sham/tDCS), per congruency condition at baseline (T1 sham phase and T3 tDCS phase), immediately after intervention (T2 sham phase and T4 tDCS phase), and 2 months after intervention (T3 sham phase and T5 tDCS phase). Differences were significant after tDCS only, with a decrease of mean response time in the incongruent condition, T4 compared to T3, lasting until follow-up, T5.

A repeated measures ANOVA revealed a significant effect for congruency on executive functioning, measured with the ANT, F(1, 92) = 63.27, *p* < 0.001, After Bonferroni’s correction, pairwise comparisons revealed a significant treatment*congruency*time interaction, F(1, 92) = 5.93, *p* = 0.017, with a significant decrease of response time after tDCS (649.22 ms, T4) compared to baseline (697.46 ms, T3) in the incongruent condition, *p* = 0.041 (see Fig. 4), lasting until follow-up (T5), *p* = 0.5. Further, the treatment*time interaction, F(2,92) = 5.14, *p* = 0.016, was significant: response times at follow-up in the incongruent condition were significantly lower after tDCS (652.33 ms) than after sham (697.46 ms). The Flanker effect (difference between incongruent and congruent mean response times) was significant at all time points in sham and tDCS treatment phases. The Stroop interference T-score improved after sham from 40 (16th percentile) to 47 (38th percentile), and declined after tDCS, from 46 (34th percentile) to 45 (31st percentile).

To summarize: trained L2 naming, untrained L1 and L2 naming, untrained L2 syntactic comprehension and repetition scores and the response time in the incongruent condition of the ANT improved after anodal tDCS only.

## DISCUSSION

We illustrated the case of Mr. G., a bilingual individual with non-fluent PPA, and the effects of tDCS applied to the cerebellum on language symptoms and two tasks for inhibitory control. We will first discuss our findings of his patterns of language decline caused by PPA, and then discuss tDCS-related effects. In short, we sound significant improvements after tDCS, but not after sham, on a trained picture naming task in L2, with generalization and CLT to untrained picture naming in his L2 and L1, as well as generalization to untrained tests of syntactic comprehension and repetition in L2. Further, tDCS led to a significant increase of performance on the ANT, but not Stroop task.

### Patterns of language decline

The languages of a bilingual aphasic person may undergo different patterns of impairment: the languages can decline in parallel, i.e., languages are impaired to a similar extent, or differentially, i.e., one of the languages is more impaired than the other.^107^ This variety of patterns is thought to be moderated by several (interacting) factors, which can be language-related, such as age of acquisition, premorbid language proficiency, use and exposure and language features.^107–110^ Other factors are related to mechanisms of language control, such as impaired inhibitory control.^8^ The way these factors impact language impairment in neurodegenerative disease, however, is still an enigma.^27,28^ At baseline Mr. G. demonstrated a noteworthy contrast in performance between L1 and L2 on the BNT, with significantly better results in L2. This discrepancy suggests a differential decline in linguistic ability across languages. However, when evaluating various subtests of the BAT, Mr. G. achieved a consistent score of 75% in both languages. This differential decline on the BNT across his languages alongside parallel decline on the BAT may reflect complex interactions between language-specific factors and proficiency level. In their retrospective review on bilingual PPA, also found heterogenous patterns of language severity across domains, echoing the complexity in Mr. G’s case. ^27^ His superior performance in his dominant language (L2) on the BNT, may indicate language dominance and proficiency effects. Furthermore, SLT was provided in the dominant language (also prior to inclusion in our study), which could contribute to greater familiarity and skill in lexical retrieval in that language. Despite differential performance on the BNT, the consistent performance across languages on the BAT suggests a generalized impairment affecting multiple aspects of language processing, despite language-specific variations in performance.

Regarding proficiency prior to diagnosis, Mr. G. indicated balanced bilingualism on the LEAP-Q questionnaire, using both languages with a similar proficiency and frequency in a variety of communicative contexts. He reported his L2 to be his dominant language, even when only learned at the age of twelve. Our findings of a relatively parallel decline of his L1 and L2 are in line with previous findings from reports of balanced bilingual speakers with PPA and AD, who also had comparable levels of impairment in their languages, as well as the study by Costa et al. (2019), who found relatively parallel decline in LvPPA participants.^14,17,20,24,27,111–114^ While there is evidence from post-stroke aphasia that age of acquisition may interact with language proficiency and dominance, our results support the suggestion that proficiency and language use may be more prominent determinants in bilingual aphasia than age of acquisition.^27,115^

Regarding bilingual language processing, findings of parallel language decline could be seen as support for the convergence hypothesis, stating that the neural substrates of language representations are shared between the languages of bilingual speakers.^116^ However, importantly, given the small amount of research on bilingual PPA, definitive conclusions remain elusive.

### Effects of cerebellar tDCS task performance

Our preliminary results are the first to indicate that tDCS applied to the cerebellum may be a valuable tool to enhance the effects of SLT in patients with LvPPA. Mr. G. received nine sessions of therapy in L2, consisting of semantic as well as phonological treatment, over the course of three weeks, combined with sham and anodal tDCS, in a within-subject controlled design. Only after anodal tDCS, but not after sham, did results improve significantly on trained picture naming (L2), untrained picture naming in L1 and L2, untrained syntactic comprehension and repetition of words and nonwords in L2, as well as on the response times in the incongruent condition of the ANT, indicating increased inhibitory control. These findings are similar to our recently published case-study on tDCS in bilingual non-fluent post-stroke aphasia.^62^ Interestingly, these findings cannot merely be described to task practice effects, as sham was given in the first treatment phase, and tDCS in the second treatment phase.

#### Language outcomes

The significant improvements seen with tDCS combined with SLT, contrast the lack of observed effects after the sham phase. In general, SFA and phonological treatment have been found to be effective in LvPPA, but the effectiveness may depend on various factors such as the specific characteristics of the therapy, individual variability in treatment response, and the severity and stage of the disease.^117,118^ In Mr. G’s case, low memory and attention scores could be factors contributing to the lack of significant findings in the sham stimulation condition. The improvements found with anodal stimulation, however, indicate that tDCS may have provided targeted neural modulation that enhanced the effects of SLT, primed neural circuits to be more receptive to subsequent cognitive interventions, or exerted synergistic effects with SLT on language processing.^119^

Our findings are in line with the literature on tDCS indicating an increased generalization of effects in post-stroke aphasia as well as PPA.^120–123^ The observed improvements in linguistic tasks such as picture naming, sentence comprehension and word repetition after cerebellar tDCS suggest a multifaceted role of the cerebellum in language processing. tDCS may have improved domain-general cognitive functions (e.g., executive control), which are crucial for language processing, thereby facilitating language rehabilitation. However, its effects may extend to specific linguistic processes affected by LvPPA. The cerebellum, through its dense structural connections with the cortical left-hemisphere language areas, likely plays a crucial role in integrating and modulating language-related networks.^67,124^ The cerebellum’s structural connections to cortical regions implicated in semantic processing may have facilitated the integration of semantic information, leading to improve retrieval of word meanings during picture naming and sentence comprehension tasks. Additionally, the cerebellum’s role in predictive processing and error detection, could have optimized phonological processing, by aiding in the anticipation of upcoming phonological sequences and detecting and correcting phonological errors, thereby enhancing word repetition accuracy.^125–127^ Syntactic comprehension may have been improved through the cerebellum’s involvement in sequencing and prediction, which are essential for parsing and interpreting sentence structures (i.e., accurate sequencing of linguistic elements and predicting upcoming syntactic features).^128,129^ Furthermore, cerebellar tDCS enhanced inhibitory control, which may have improved test performance by for instance suppression of irrelevant linguistic information.^130^ The contributions to verbal working memory and phonological processing (e.g., Mariën et al.^69^ Marvel et al.^131^ Peterburs et al.^132^) is also relevant to its involvement in language processing and likely contribute to the observed enhanced performance on naming, repetition, and sentence comprehension tasks. ^133–136^ By enhancing working memory function, cerebellar tDCS may improve the Mr. G.’s ability to hold and manipulate linguistic information, leading to more efficient language comprehension and production.

Further, we found CLT effects for the untrained picture naming task, namely the Boston Naming Test (BNT) in French (L1) and Dutch (L2). In post-stroke aphasia, factors influencing the potential for CLT are thought to be: pre-morbid and post-morbid proficiency in each language, structural similarities and differences across the languages, word type (cognate/noncognate) and cognitive control.^33^

Regarding pre-and post-morbid proficiency, our results align with evidence that balanced bilingualism contributes to CLT.^137^ In cases of unbalanced bilinguals, transfer is thought to be more likely to occur from the less proficient language to the dominant language.^138,139^ This may again be explained by the hypothesis by Green:^116^ As Mr. G. was highly proficient in both languages to an equal extent, similar neural structures may be involved for processing both languages, particularly in tasks that require lexical-semantic processing.^137^ Further, intact executive functioning (or cognitive control) is proposed to contribute to CLT.^33^ The cognitive control circuit is suggested to be pivotal for adequate selection and/or inhibition of either language considering a given communication context and has been proposed to be a contributing factor to patterns of language impairment, but also the possibility of CLT. As such, it is plausible that tDCS applied to the cerebellum may have helped to facilitate CLT by enhancing inhibitory control.^107^

For completeness, we would like to add our findings of cognate status effects on picture naming results. There has been discussion about the use of cognate status in treatment of bilingual aphasia. The use of cognates has been proposed to have a faciliatory effect on lexical retrieval and is by some thought to be essential to increase probability of CLT.^140–142^ This would occur through co-activation of both languages through the shared phonological properties of a target word. Others state that this could lead to interference, definitely in the presence of language control deficits.^143^ In our current study, all picture naming tests include Dutch-French cognate items (BNT: 26%, object naming sham: 25%, object naming tDCS: 19%), which allows closer inspection on this matter. On the personalized object naming tests, we don’t see an effect of cognate status, but on the BNT, Mr. G.’s increased performance for naming picture items was present for cognates, but noticeably larger for non-cognates. Importantly however, cognates and non-cognates of the BNT are not matched for word frequency or length, and the cognate words generally consisted out of more syllables than the non-cognates, so the phonological difficulty may have impacted results. We can therefore only carefully state that tDCS has also contributed to facilitation of naming of non-cognates, and is not limited to cognates, which offers possibilities for treatment and future research.

#### Inhibitory control

To evaluate executive functioning of Mr. G., and the effects of cerebellar tDCS thereon, we performed the Attention Network Test and Stroop test. Executive function is examined by comparing the response times for incongruent conditions to those for incongruent conditions.

His baseline results indicated a very effective inhibitory control performance. On the ANT, with an average response time of 660.02 ms on the incongruent condition and a Flanker score of 76.07 ms, he scored remarkably better than the healthy bilingual group in Mishra et al. where participants (average age of 57) had an average response time of 880.95 ms in the incongruent condition and a Flanker score of 126.41 ms (we did not find published data of ANT results in PPA).^144^ On the Stroop test, his raw interference score was 77 s at baseline, with a T-score of 40 (16th percentile).^106^ In PPA, we did not find normative data on the Stroop test, but the average interference score of all studies included in our published meta-analysis is 120 s (monolingual PPA, average age of 64).^51^ Mr. G.’s absence of deficits in inhibitory control may have favored therapy effects in both languages, and thus CLT. Additionally, we found increased inhibitory control performance after tDCS on the ANT, as indicated by a decreased response time in the incongruent condition after tDCS, lasting until follow-up, but not after sham stimulation.^8^ Thus, tDCS effects may also have been mediated through an increase in cognitive control. This finding of language improvements going hand in hand with increased (nonverbal) inhibitory control, could speak in favor of domain-general cognitive control playing a role in language. His Stroop interference score on the contrary, are not in line with his ANT result. Here, Mr. G’s interference score decreased after sham, and increased after tDCS. As the higher score of the first assessment is the ‘odd one out’, and his score is very similar throughout all other time points, the change in performance here may be due to a practice effect. Practice effects are often larger in the first phase, as task familiarization is larger when commencing the second phase. Another contributing factor to these discrepancies may lie in the divergent cognitive demands of the tasks. While both tasks engage inhibitory control regions, the Stroop task additionally recruits language processing areas, potentially influencing tDCS effects.^145,146^ Nonetheless, this is not consistent with our language-related effects, as these did improve after tDCS, and not after sham. This complexity mirrors mixed findings from prior research on cerebellar tDCS effects in healthy cohorts, underscoring the specificity of stimulation effects to specific tasks and difficulty levels.^147–149^ Furthermore, dementia is known to lead to larger intra-individual variability in Stroop task performance.^150^ Given this variability and compromised neural circuitry in PPA, the sensitivity of the Stroop task to detecting the effects of cerebellar tDCS on complex inhibitory control processes may be limited, necessitating cautious interpretation of tDCS effects in this case-study.

### Limitations and future directions

The main limitation of this study is that it is a case-study, so results are preliminary and have to be validated in larger groups. As bilingual primary progressive aphasia is quite rare, small sample sizes are unfortunately to be expected in this field. We are recruiting patients to conduct the study on a larger number of patients, in order to increase generalizability of effects. Additionally, we found differential effects of cerebellar tDCS on the ANT and Stroop task. Possibly, the Stroop task is not optimal for evaluating the effects of cerebellar tDCS in LvPPA, however; future work may elucidate on this. The integration of multiple baseline assessments might offer a viable approach to mitigate variability, provided it is practical to implement in the protocol. Regarding language testing, an uneven allocation of test items across languages on the BAT hindered our ability to perform statistical comparisons between them. For certain language combinations, supplementary measures or adjustments may be necessary to ensure an equitable assessment across languages. In general, the results of this study indicate call for further exploration of the effects of tDCS applied to the cerebellum.

Future research could investigate optimal cerebellar tDCS application for LvPPA. Potential avenues include exploring standalone cerebellar stimulation or combining it with targeting other brain regions. Personalized stimulation protocols tailored to individual symptoms and response could enhance therapeutic benefits. Regarding future studies, we hypothesize here that one possible explanation of our results is the contributions of the cerebellum to phonological processing. However, there is still no clear understanding of how the cerebellum contributes to phonological processes, and to which subprocesses specifically. Further, little is known on breakdown of phonological functions in PPA.^151^ Future research may elucidate on the role of the cerebellum in language processing, in a population with or without PPA.

### Conclusion

We have applied anodal tDCS to the right posterolateral cerebellum combined with speech and language therapy in a bilingual patient with logopenic PPA. Stimulation led to greater effects than sham, with regard to the trained task. Further, tDCS led to generalization of effects to untrained tasks, the untrained language, and a task for inhibitory control. The observed enhancements in linguistic tasks following cerebellar tDCS underscore the pivotal role of the cerebellum in language processing, with implications for both domain-general cognitive functions and specific linguistic processes affected by LvPPA. By modulating neural networks involved in semantic, phonological, syntactic, and control aspects of language processing, cerebellar tDCS holds promise as a targeted intervention for improving language function in individuals with LvPPA, highlighting the potential of this approach for advancing language rehabilitation strategies.

## AUTHOR CONTRIBUTIONS

Silke Coemans (Conceptualization; Data curation; Formal analysis; Investigation; Methodology; Project administration; Visualization; Writing – original draft; Writing – review & editing); Vânia De Aguiar (Writing – review & editing); Philippe Paquier (Writing – review & editing); Kyrana Tsapkini (Writing – review & editing); Sebastiaan Engelborghs (Writing – review & editing); Esli Struys (Conceptualization; Funding acquisition; Project administration; Supervision; Writing – review & editing); Stefanie Keulen (Conceptualization; Funding acquisition; Project administration; Supervision; Writing – review & editing)

## Supplementary Material

Supplementary Table

## Data Availability

The data supporting the findings of this study are available within the article and/or its supplementary material.
